# In-situ sequencing reveals the effect of storage on lacustrine sediment microbiome demographics and functionality

**DOI:** 10.1186/s40793-022-00400-w

**Published:** 2022-01-31

**Authors:** Richard K. Tennant, Ann L. Power, Sara K. Burton, Norman Sinclair, David A. Parker, Richard T. Jones, Rob Lee, John Love

**Affiliations:** 1 Geography, College of Life and Environmental Sciences, The University of Exeter, Exeter, EX4 4RJ UK; 2 Biosciences, College of Life and Environmental Sciences, The University of Exeter, Exeter, EX4 4QD UK; 3 The Orkney Brewery, Quoyloo, Stromness, Orkney, KW16 3LT UK

**Keywords:** sedDNA, sedRNA, Storage, Holocene sediments, Microbiome, ONT MinION sequencing, Bioinformatics

## Abstract

The sediment microbiome is a demographically diverse and functionally active biosphere. Ensuring that data acquired from sediment is truly representative of the microbiome is critical to achieving robust analyses. Sample storage and the processing and timing of nucleic acid purification after environmental sample extraction may fundamentally affect the detectable microbial community and thereby significantly alter resultant data. Direct sequencing of environmental samples is increasingly commonplace due to the advent of the portable Oxford Nanopore MinION sequencing device. Here we demonstrate that storing sediment subsamples at − 20 °C or storing the cores at 4 °C for 10 weeks prior to analysis, has a significant effect on the sediment microbiome analysed using sedimentary DNA (sedDNA), especially for *Alpha*-, *Beta*- and *Deltaproteobacteria* species. Furthermore, these significant differences are observed regardless of sediment type. We show that the taxa which are predominantly affected by storage are *Proteobacteria*, and therefore recommend on-site purifications are performed to ensure an accurate representation of these taxa are observed in the microbiome. Comparisons of sedimentary RNA (sedRNA) analyses, revealed substantial differences between samples purified and sequenced immediately on-site, samples that were frozen before transportation, and cores that were stored at 4 °C prior to analysis. Our data therefore suggest that a more accurate representation of the sediment microbiome demography and functionality may be achieved by environmental sequencing as rapidly as possible to minimise confounding effects of storage.

**Supplementary Information:**

The online version contains supplementary material available at 10.1186/s40793-022-00400-w.

## Background

Lacustrine sediments are an excellent archive of microbial communities [1]. Direct sequencing of DNA purified from sediments (sedDNA) is increasingly used to provide a census of the microbial biodiversity in different habitats [2–5] and RNA (sedRNA) sequencing can be used to infer microbiome activity and function [6]. Validation of the sediment microbiome demographics is therefore fundamental to environmental metagenomics and for robust paleoclimate reconstructions [7]. Additionally, accurate classification of the microbial functionality will increase our understanding of the fundamental processes of the biosphere [8], and the effects of environmental change and may also provide the material and inspiration for potential biotechnological applications [9].

Metagenomic analysis of sedDNA has become a frequently utilised and an accepted method for ecological and palaeoenvironmetal reconstructions [10] and enables the identification of organisms not readily identified using conventional microscopic techniques [1, 11]. However, metatranscriptomic analysis of sedRNA is comparatively still a developing field of research, due to the complexity and diversity of soil ecosystems [8, 12].

Industry and research institutions worldwide have extensive libraries of stored sediment cores. The effect of this storage on the microbial demography [13, 14] and functionality [15] of marine sediments has been investigated and suggests that storage has a detrimental effect on the sediment microbiome when compared to fresh samples, but such analyses have not been extensively performed on other sediment types, notably from extant or ancient lacustrine environments which integrate a wide and potentially diverse catchment [1], and may contain more diverse microbiomes than the more stable marine habitat. Typically, molecular analysis of lacustrine sediments is performed up to 2 months after core extraction [16]. Whilst it has been shown that short term (< 14 days) storage has a limited effect on demographics when analysing the sedDNA [13], it is unclear what changes are observed in the demography and functionality when lacustrine sediments are stored for longer at 4 °C, mimicking typical handling of lacustrine sediment cores prior to analysis. Recommendations recently outlined for sedDNA core extraction and subsequent DNA analysis [10], highlighted the necessity to store cores in conditions similar to their originating environments. However these maybe difficult to replicate due to specific below ground conditions, such as, oxygen concentration, temperature gradients and moisture content. Furthermore, storage at − 20 °C has been recommended for sub-sampled sediments to minimize cross-contamination and sample degradation, but the effect of this storage is unknown. A further complication is that it is not always possible to transport soil and sediment samples internationally between field sites and laboratories due to import regulations. It is not practical to extract and transport sediment cores longer than 1 m, therefore a multiple dual coring approach is employed to extract a complete record [17], whereby two coring holes, typically 30 cm apart, are alternatively sampled at the required depths to minimise sediment disruption and contamination. Overlapping sections of core are extracted to ensure a complete sediment record is achieved.

To investigate the changes of the microbiome structure and functionality in sediment archives following removal and storage, we extracted two adjacent (1 m apart) sediment cores from an ancient lake site, sub-sampling one core immediately and sequencing the sedDNA and sedRNA on site using the Oxford Nanopore MinION. Additional subsamples were extracted from the same core and stored at − 20 °C. Subsequent sedDNA and sedRNA analysis was performed upon return to the laboratory one week later. The second sediment core was wrapped immediately and also analysed on return to the laboratory and after 10 weeks storage at 4 °C.

## Materials and methods

### Core extraction and storage

**Fig. 1:**
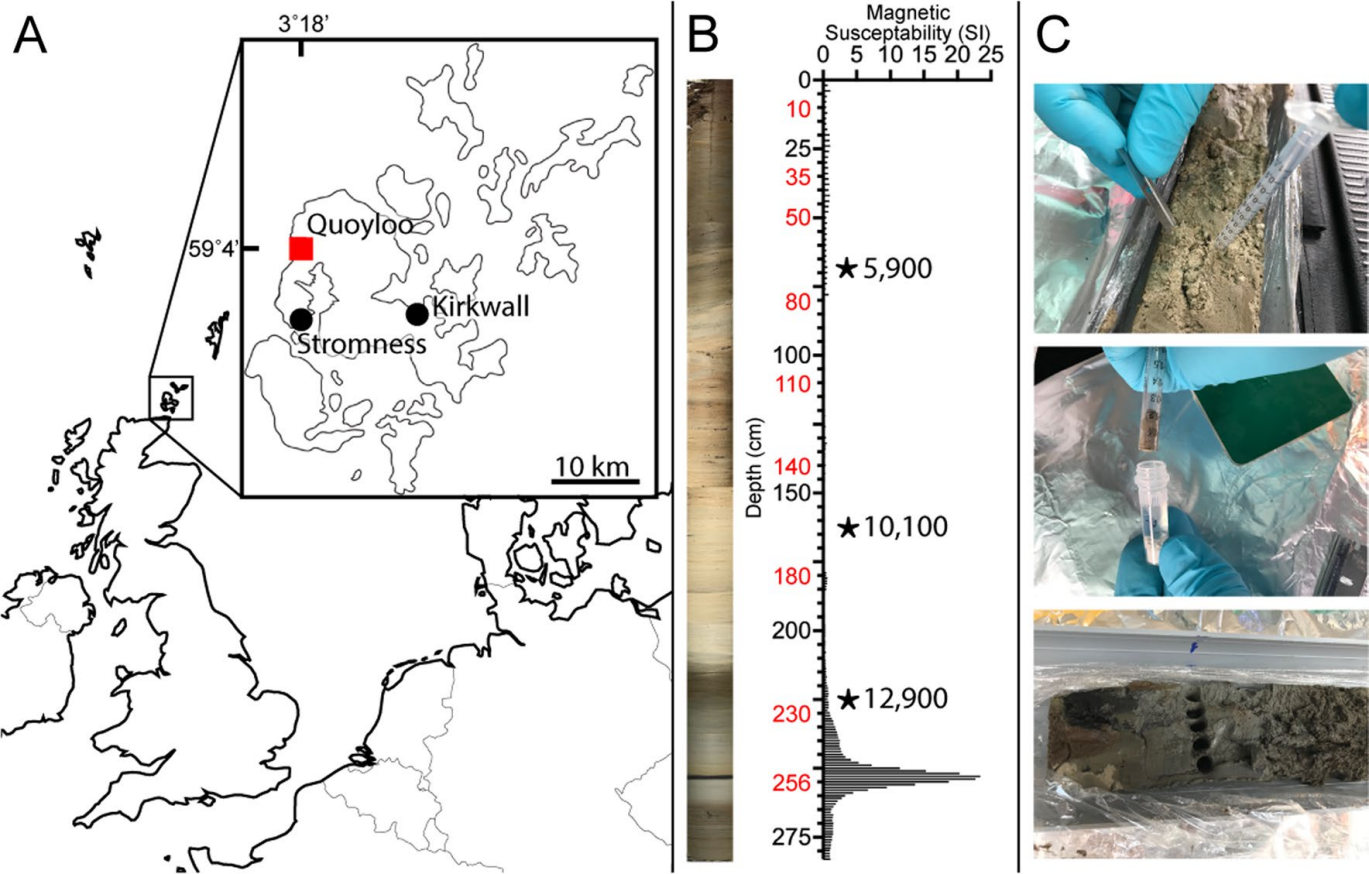
Location of Quoyloo Meadow and sediment analysis. **A** Map highlighting the location of Quoyloo Meadow, on the Orkney Islands. **B** Composite image of the extracted core and magnetic susceptibility results of core A. Note the peak in magnetic susceptibility identified at 255 cm, which corresponds with a black band present in the sediment at the same depth. ^14^C radiocarbon dates from Bunting [18], are indicated with a star at equivalent sediment depths. Ages are reported in cal yr BP. **C** Example images of sediment core sub-sampling; Top panel, image demonstrating how the sediment core was sub-sampled; Centre panel, image showing how extracted sub-samples were transferred to a lysis tube; Lower panel, image displaying all 5 sub-sampling locations within an individual sediment horizon

Two adjacent (1 m apart) complete 2.8 m sequences of sediment deposits were extracted using a 5 cm diameter Russian corer from Quoyloo Meadow, Orkney, Scotland (59° 4′ 3.72″ N, 3° 18′ 42.84″ W) (Fig. 1) a Holocene, infilled carbonate lake [18] which was located adjacent to ‘The Orkney Brewery’. Cores were recovered in 0.5 m intervals, allowing for a 10 cm overlap between multiple sampling depths and the corer was cleaned between samples. Core A was subsampled in the field before being sealed with clingfilm. Core B was immediately sealed in clingfilm. Cores were transported for 36 h, from Orkney to The University of Exeter at ambient temperature (≈ 15 °C). Subsamples were transported from Orkney to The University of Exeter at − 20 °C using a 12 V freezer. Cores A and B were stored at 4 °C immediately upon arrival to the laboratory and Core A subsamples stored at − 20 °C. After 10 weeks, identical subsampling, purification and sample preparation were performed on Core B.

### Magnetic susceptibility

Core A was scanned using a Bartington MS2C core-logging sensor to determine volume specific magnetic susceptibility [19].

### Core sub sampling

All subsampling was performed using a single use, sterile 1 ml syringe with the tip removed. 10 depths (10, 35, 50, 58, 80, 110, 140, 180, 230 and 256 cm) were chosen based upon visual inspection of the transitions in sediment composition. For core A, 4 subsamples were taken at each depth. 1 for onsite sedDNA analysis, 1 for onsite sedRNA analysis, 1 which was stored at − 20 °C for later sedDNA analysis, 1 which was stored at − 20 °C for later sedRNA analysis. No cryoprotectant or stabilising agent was added to the subsamples. For Core B, after 10 weeks storage at 4 °C, 2 subsamples were taken at each depth, 1 for sedDNA analysis, 1 for sedRNA analysis. At each sampling depth a single sample was used for the designated analysis. Samples taken from Core A for onsite analysis were designated as treatment ‘As’, those subsampled immediately and analysed later treatment ‘Af’ and samples taken from sediment which was stored for 10 weeks at 4 °C before analysis, are defined as treatment ‘B’.

### Field laboratory setup

A field laboratory was established in the rear of a sport-utility (SUV) type vehicle. A refrigerated centrifuge, PCR thermocycler, rotating incubator and MPBio FastPrep 24 was transported to the site so that the same equipment was used for the purifications from all 3 different storage conditions and exclude effects of instrument variability. A 12 V − 20 °C freezer was used to transport reagents, purified samples and frozen sediment samples. Mains power (240 V, 50 Hz) was provided onsite by The Orkney Brewery.

### sedDNA and sedRNA purification

sedDNA was purified using the MPBio FastDNA for Soils kit (MP Biomedicals, USA) following the manufacturer’s instructions with a final elution volume of 100 µl. sedRNA was purified using the MPBio FastRNA Pro Soil Direct kit (MP Biomedicals, USA) following the manufacturer’s instructions with a final elution volume of 50 µl. Purified sedDNA and sedRNA was quantified using the Qubit assay (Thermo-Fisher Scientific, UK). Two negative controls were performed without sediment.

### sedDNA repair and sequencing library preparation

Purified sedDNA was repaired using PreCR Repair Mix (New England Biolabs, USA). Repaired sedDNA sequencing libraries were prepared using the low input genomic DNA by PCR Barcoding (SQK-LWB001) method (Oxford Nanopore Technologies, UK).

### sedRNA poly A tailing and sequencing library preparation

A poly-A tail was fused to the 3’ terminus end of the purified sedRNA using *E. coli* Poly(A) polymerase (New England Biolabs, USA). Poly-A tailed RNA sequencing libraries were prepared using the 1D PCR barcoding cDNA (SQK-LSK108) method (Oxford Nanopore Technologies, UK).

### sedDNA and sedRNA sequencing

Prepared sedDNA and sedRNA libraries were sequenced independently using a MinION with a FLO-MIN106 R9.4 flowcell (Oxford Nanopore Technologies, UK). Separate sequencing flowcells were used for each of the storage conditions.

### Bioinformatic analyses

Sequence data analysis was performed using a local server containing 32 3.1 GHz CPUs and 256 Gb RAM. The system was installed with the Fedora v.21 Linux operating system. Sequence reads were basecalled using Albacore v2.3.3 and verified using porechop v0.2.3. Singletons were not removed and data was not normalised before classification. sedDNA and sedRNA sequences were taxonomically classified using DIAMOND v0.9.19.120 [20] with a frameshift value of 15. Taxonomies were visually represented using MEGAN v6.11.1 [21]. Taxonomic heatmaps were generated from data exported from MEGAN using R v3.6.0 with ggplot2 package. PCoA analysis was performed with Bray–Curtis dissimilarity using MEGAN v6.11.1. Stacked bar charts were constructed using classified data exported from MEGAN and visualised using GraphPad Prism v9.2. Statistical analyses were performed using SAS JMP Pro v16 and GraphPad Prism.

## Results

### Core analysis

The Quoyloo meadow sediment cores A and B transition from being predominantly organic in the upper 75 cm of the core to a mainly carbonate composition from 75 to 215 cm. The lower section of the cores, from 215 to 280 cm were comprised of clay sediment with a prominent 1 cm thick black band identified at 255 cm. The magnetic stratigraphy for Core A (Fig. 1) correlated with previous analysis from Quoyloo Meadow [18, 22], with relatively high magnetism in the upper 60 cm of the core. A reduction in magnetism coincided with carbonate substrate and magnetic susceptibility increased at 200 cm as the sediment transitioned to clay. A magnetic peak at a depth of 180 cm, corresponded to a peak identified by Bunting [18] and Timms [22] at a sediment depth of 160 cm, which they attributed to Saksunarvatn Ash tephra (10,257–10,056 cal yr BP). A unique feature of both cores A and B from Quoyloo Meadow, was a distinct 1 cm thick black band at a sediment depth of 255 cm. This band occured at the same depth in each of our cores, indicating homogeneity between the cores. Magnetic susceptibility was not measured for core B. The band corresponds to a prominent magnetic maximum an order of magnitude greater than the tephra peak at a depth of 180 cm and may reflect the global black mat phenomenon observed in Younger Dryas boundary layers in sediment archives worldwide [23].

### Demography of the sediment microbiome by sedDNA analysis

sedDNA was purified and sequenced from each of the treatments—As, Af and B (Table 1), using the Oxford Nanopore MinION to determine the demography of the environmental microbiome. Sequenced sedDNA was taxonomically classified using DIAMOND software and data was visually represented using MEGAN (Table 2). Rarefaction analysis of each of the storage conditions (Additional file 1: Fig. S1) indicates that a sufficient depth of sequencing was achieved for reliable analysis.

**Table 1:** Storage and subsampling strategy for cores A and B

Treatment name	Core A		Core B
	As	Af	B
Subsampled	Immediately	Immediately	After 10 weeks
Core storage temp (°C)	–	–	4
Subsample storage temp (°C)	–	− 20	–
Core storage time (weeks)	–	–	10
Subsample storage time (weeks)	–	1	–
sedDNA sequenced	On site	In lab	In lab
sedRNA sequenced	On site	In lab	In lab

**Table 2:**
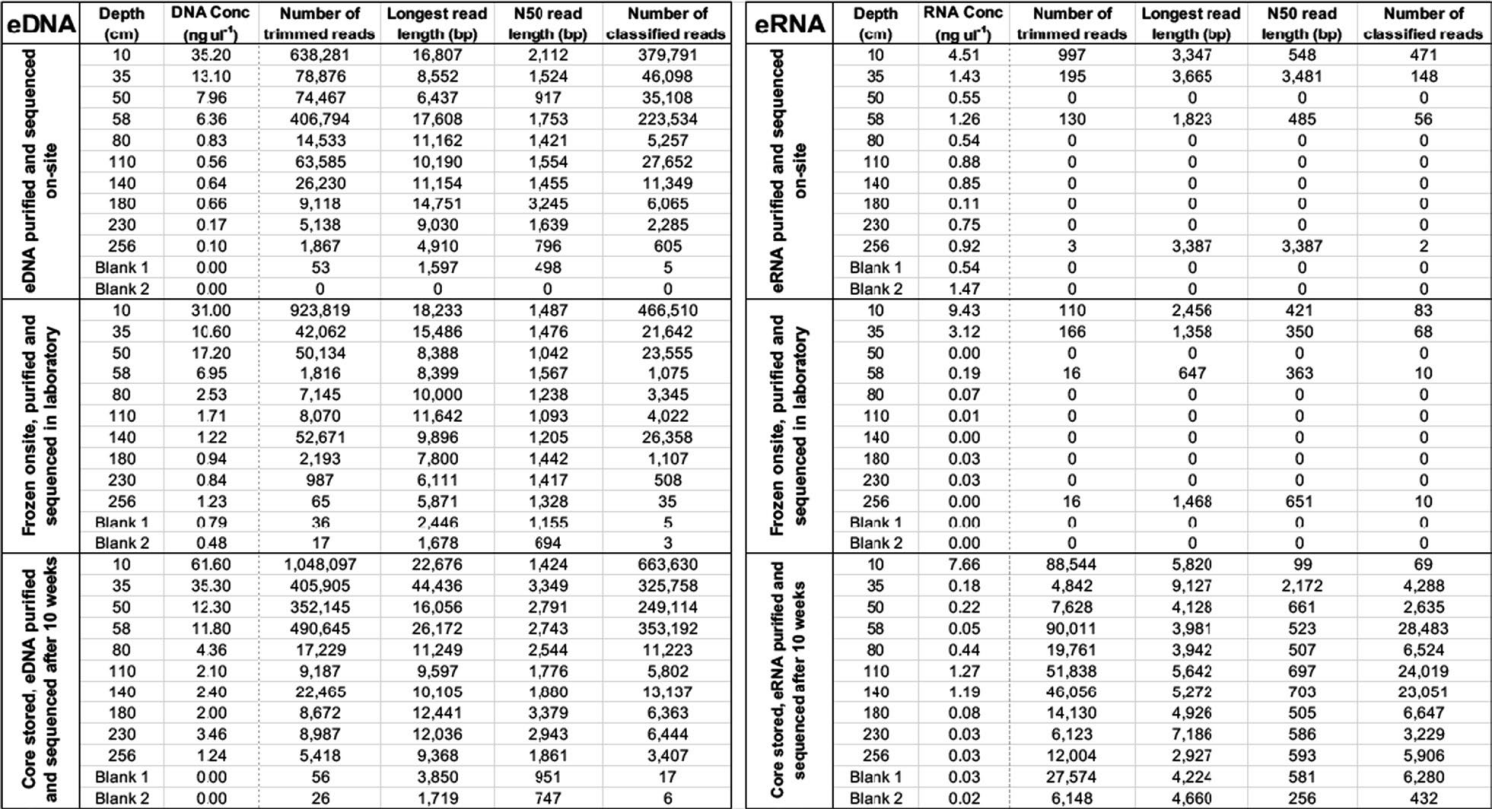
Summary of sedDNA and sedRNA purified from Quoyloo meadow sediment and the resultant sequence data

**Fig. 2:**
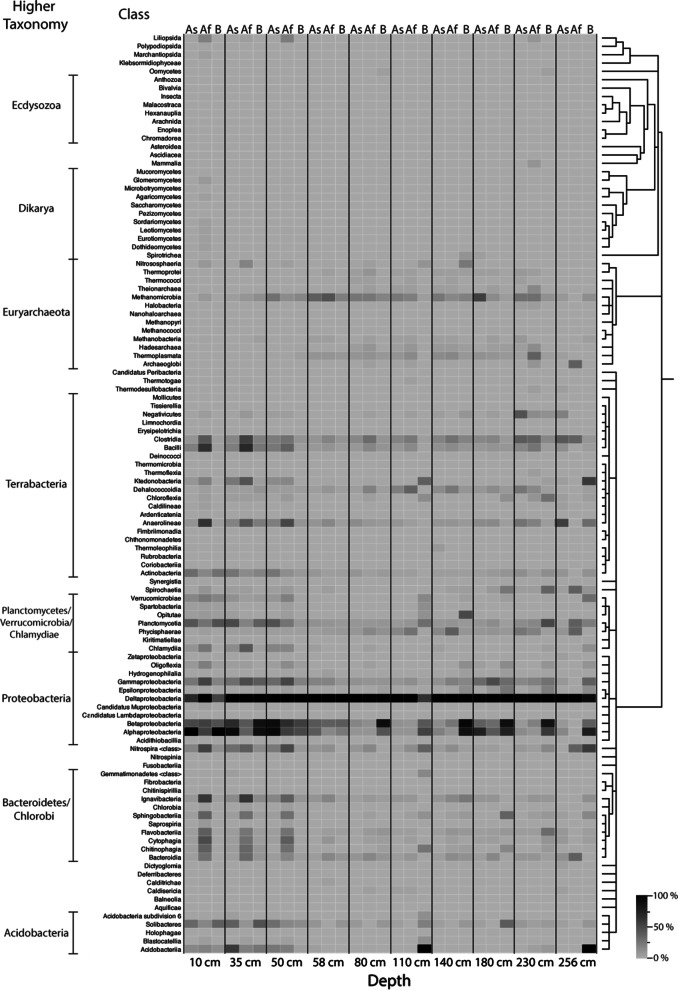
Heatmap representing the taxonomic classifications of sedDNA samples at each condition. Heatmap of the classified taxa at the taxonomic rank of Class displayed using percentage classification. Darker shades denote higher percentages of classified sedDNA sequence reads to that assigned taxa. Condition As—sediment subsampled onsite and sequenced immediately, Af—sediment subsampled onsite, frozen at − 20 °C and sequenced later and B—sediment stored at 4 °C

Abundances of the classified taxa for each sample and storage condition were displayed as a heatmap at the Class taxonomic level (Fig. 2 and Additional file 1: Table S1). The most obvious finding between each storage condition is the consistent abundance of the Class Deltaproteobacteria throughout the entire sediment core. A high abundance of Alphaproteobacteria and Betaproteobacteria in sediment depths 0–58 cm can be observed for all conditions, however, below 80 cm this dominance is primarily identified in samples from condition B. In relatively recent sediments, 0–58 cm, a higher abundance of the Bacteroidetes and Chlorobi Phyla, was observed for condition Af in comparison to conditions As and B which do not exhibit the same levels of abundance. A similar disparity was observed with Clostridia and Bacilli (Phylum Terrabacteria) which consistently have a higher abundance in condition Af to a depth of 180 cm compared to samples from condition As and B. The abundance of the class Methanomicrobia is consistently higher in conditions As and Af in sediment depths from 50 to 230 cm, compared to condition B. In the most recent (< 50 cm), and deepest core sections (> 230 cm) there is a higher abundance of Methanomicrobia found in condition B. In the top 50 cm layer of the sediment, more Planctomycetia were identified in sediment that was not frozen, *i.e.* in conditions As and B. However, in the sediment, there are more Chlamydiia identified in condition Af, which was frozen, than conditions As and B, which were analysed immediately after subsampling. The abundance of Chlamydiia is low in sediment below 50 cm for each condition. Notably, Planctomycetia exhibits higher abundance in condition B at 110 and 230 cm and a greater abundance at a depth of 256 cm in conditions Af and B, than those of condition As.

### Effect of storage on the demography of the most abundant taxa

**Fig. 3:**
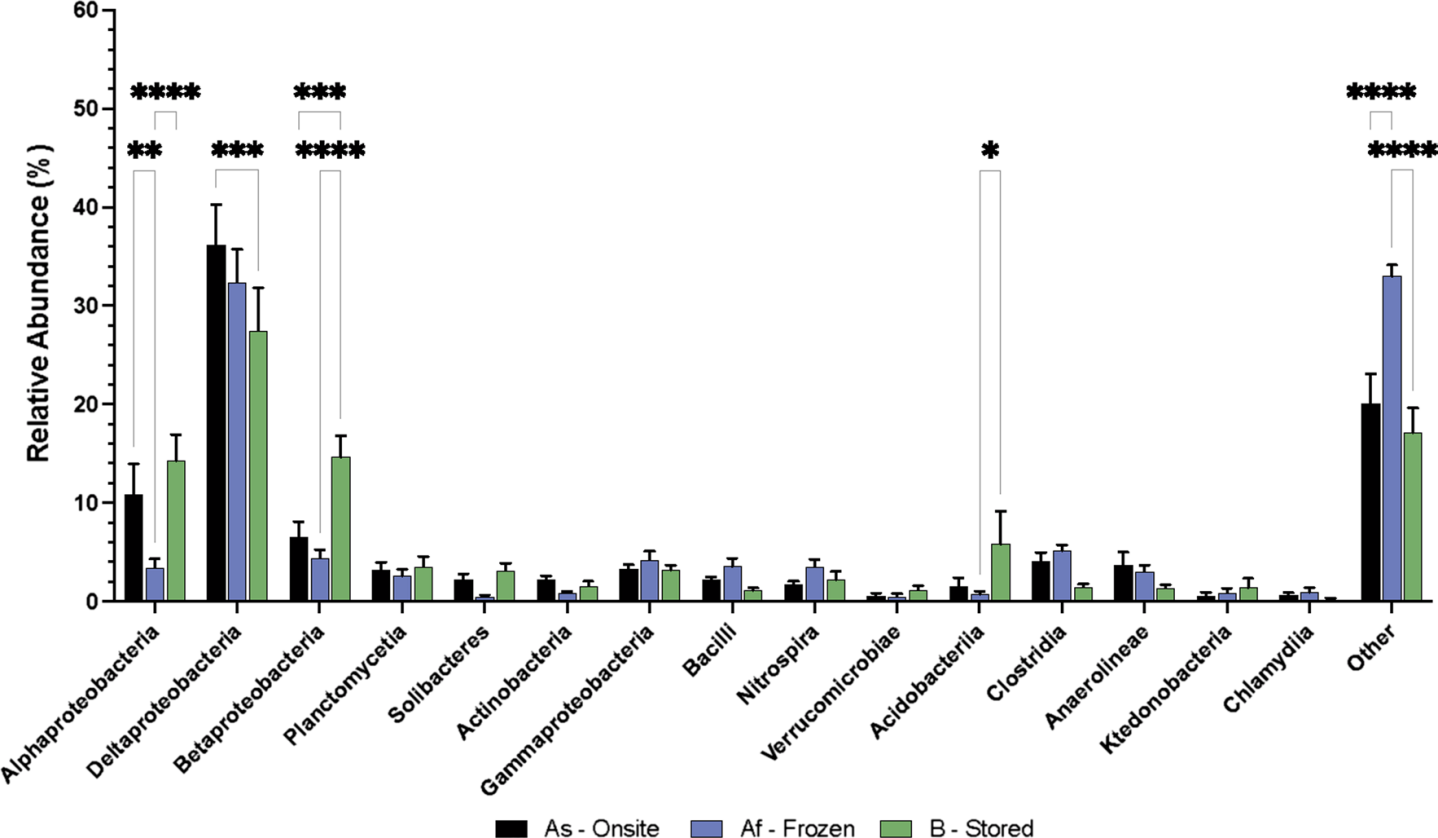
Taxonomic classification of the sediment microbiome observed under each of the storage conditions. Onsite subsampling and sequencing (black bars), frozen subsamples taken onsite and later (36 h?) sequenced in the laboratory (blue bars) and core stored for 10 weeks at 4 °C prior to subsampling and sequencing (green bars). Error bars indicate standard error, n = 10. Asterisks indicate significant (P =  < 0.05) results of pairwise comparisons using Tukey multiple comparison test; number of asterisks indicate level of statistical significance

The 15 most abundant taxa at class taxonomic rank from condition As (Alphaproteobacteria, Deltaproteobacteria, Betaproteobacteria, Planctomycetia, Solibacteres, Actinobacteria, Gammaproteobacteria, Bacilli, Nitrospira, Verrucomicrobiae, Acidobacteriia, Clostridia, Anaerolineae, Ktedonobacteria and Chlamydiia), were selected to facilitate the comparison between sample storage conditions and timing of environmental nucleic acid purification. The total abundance of all other taxa identified within the samples was categorised by ‘Other’. These 15 taxa were used as a baseline against which taxonomic classifications from Core Af and B were compared. To establish the holistic effect of core storage on the sediment microbiome, each of the sampled depths were treated as a replicate of the individual treatment (Fig. 3). A 1-way ANOVA for each of the selected taxa was performed to determine if there was a significant difference in their abundance between each of the treatments. Subsequent pairwise comparisons for the individual taxa were conducted to identify significant differences between each of the treatments (Fig. 3).

Throughout the entire core the most abundant taxon observed in each of the three treatments was Deltaproteobacteria, with 36% of the microbiome in treatment As being classified as Deltaproteobacteria. The ANOVA revealed there was no overall significant difference (P = 0.3033) between the treatments for the abundance of Deltaproteobacteria. However, the pairwise comparison revealed that was is a significant difference (P = 0.0002) between treatments As and B. The Alphaproteobacteria comprise 10% of the sediment microbiome identified in treatment As and 14% of the microbiome identified in treatment B. The abundance of Alphaproteobacteria was reduced to 3% when the subsamples are frozen in treatment Af. Overall storage treatments have a significant effect (P = 0.0104) on the abundance of Alphaproteobacteria and the pairwise comparisons showed a significant difference between treatments As and Af (P = 0.0016) and treatments Af and B (P =  < 0.0001). Furthermore, there was a significant difference (P = 0.0002) in the abundance of Betaproteobacteria when stored in the different conditions, with 6% identified in As, 4% in Af and an increase in abundance to 14% observed in treatment B. The pairwise comparison reveals there was a significant difference between treatments As and B (P = 0.0005) and treatments Af and B (P =  < 0.0001). The ANOVA showed there were significant statistical differences between the treatments for Solibacteres (P = 0.0058), Actinobacteria (P = 0.0475), Bacilli (P = 0.0052) and Clostridiia (P = 0.0007), however, the pairwise comparison between the treatments for these taxa did not establish any significant differences. Moreover, the ANOVA showed that there was no significant difference in the abundance of Acidobacteria; however, the pairwise comparison revealed a significant difference (P = 0.458) in the abundance of Acidobacteria found between treatments Af and B. There were no significant differences (P =  > 0.05) observed in the ANOVA or pairwise comparisons for Planctomycetia, Gammaproteobacteria, Nitrospira, Verrucomicrobiae, Anaerolineae, Ktedonobacteria and Chlamydiia.

**Fig. 4:**
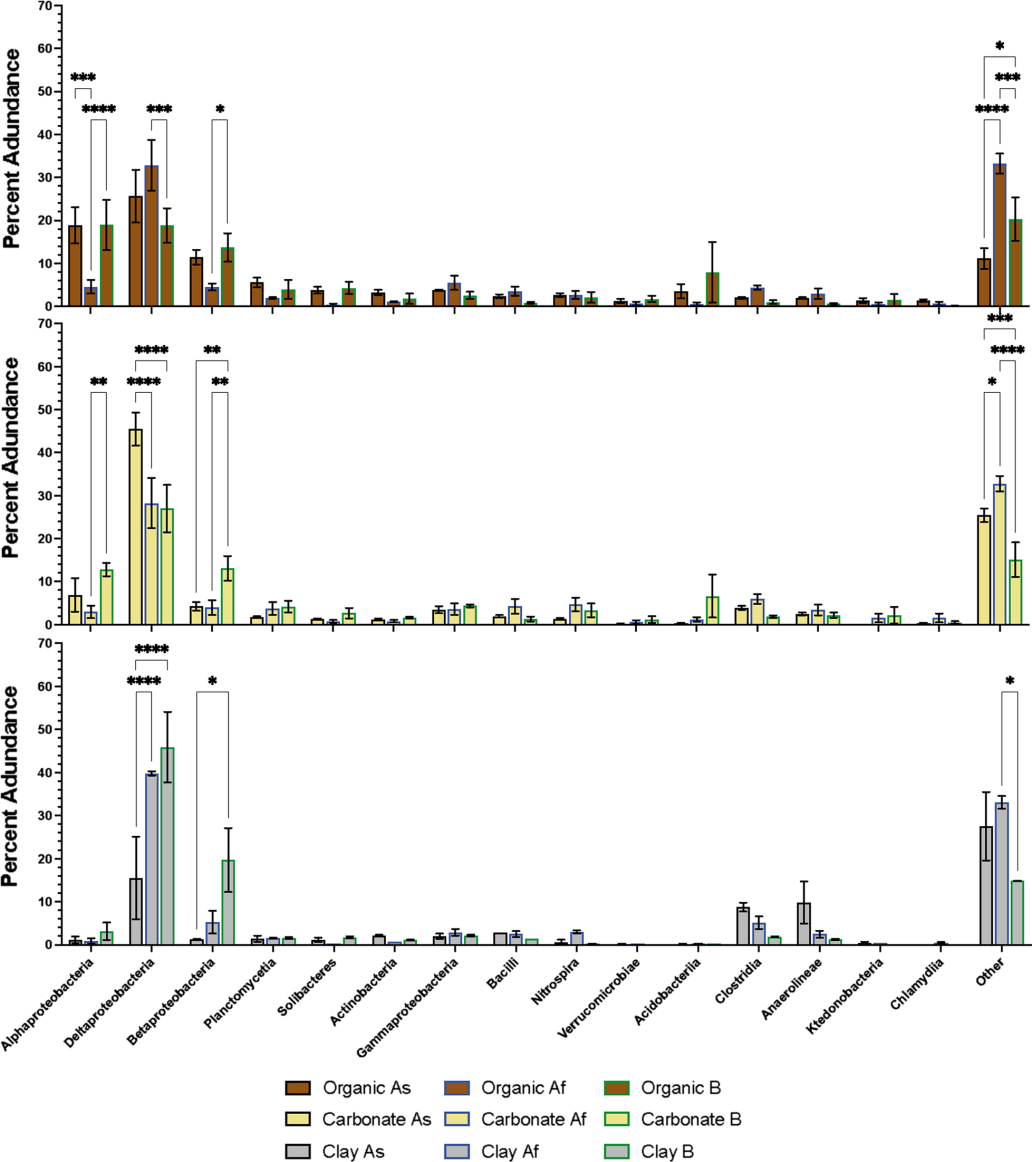
Taxonomic classification of the observed microbiome under each of the storage conditions separated by predominant sediment type. Sediment core separated by predominant composition; Top—Organic (brown bars), Middle—Carbonate (beige bars) and Bottom—Clay (grey bars). Onsite subsampling and sequencing (black borders), frozen subsamples taken onsite and later sequenced in the laboratory (blue borders) and core stored for 10 weeks at 4 °C prior to subsampling and sequencing (green borders). Error bars indicate standard error, n = 10. Asterisks indicate significant (P =  < 0.05) results of pairwise comparisons using Tukey multiple comparison test; number of asterisks indicate level of statistical significance

The sediment stratigraphy from Quoyloo meadow was comprised of three distinct sediment types, with organic (0–75 cm), carbonate (75–215 cm) and clay (215–280 cm) regions observed (Fig. 1), reflecting changing environmental conditions over time, under which these sediments were deposited [18]. The abundance of the 15 most identified taxa in treatment As were statistically analysed using a 1-way ANOVA and pairwise comparisons to establish the effect of storage on the microbiome (Fig. 4) for each sediment type.

Within the organic fraction (0–75 cm) of the cores, Alphaproteobacteria accounted for 19% of the microbiome in condition As and B. However, when subsamples are frozen and later sequenced in condition Af, the abundance of Alphaproteobacteria reduced to 5%. Overall, there was not a significant difference (P = 0.0654) between the treatments but the pairwise comparisons did reveal a significant difference (P = 0.0001) between treatment As and Af and treatments Af and B (P =  < 0.0001). The abundance of Deltaproteobacteria was 26% in treatment As, 33% when subsamples were frozen in treatment Af and 19% in treatment B. The ANOVA revealed no significant difference (P = 0.2434) between the treatments and the abundance of Deltaproteobacteria, however the pairwise comparisons highlighted a significant difference (P = 0.0002) between treatments Af and B. The ANOVA revealed a significant difference (P = 0.0386) in the abundance of Betaproteobacteria under each of the treatments. Furthermore, the pairwise comparisons showed a significant difference (P = 0.0189) between treatments Af and B for the abundance of Betaproteobacteria. The ANOVA indicated that there was a significant difference between the treatments in the abundance of Solibacteres (P = 0.0355) and Clostridia (P = 0.0004), however the pairwise comparisons did not reveal any significant differences between the treatments for these taxa. There was no significant difference (P =  > 0.05) observed between the treatments for the other highlighted taxa throughout the organic fraction of the cores.

The carbonate rich section of the cores (75–215 cm) were dominated by Deltaproteobacteria, which accounted for 46% of the microbiome in treatment As, 28% in treatment Af and 27% in treatment B. The pairwise comparisons showed a significant difference in the abundance of Deltaproteobacteria between treatments As and Af (P =  < 0.0001) and As and B (P =  < 0.0001) but overall, the ANOVA showed there was not a significant difference (P = 0.0554) between the treatments and the abundance of Deltaproteobacteria. Similarly, for Alphaproteobacteria there was a significant difference (P = 0.0015) in the abundance observed between treatments Af and B, but no overall significant difference (P = 0.0637) was identified by the ANOVA. However, the ANOVA did reveal a significant difference (P = 0.0168) in the abundance of Betaproteobacteria between the three treatments, with the pairwise comparisons highlighting a significant difference between treatments As and B (P = 0.0049) and treatments Af and B (P = 0.0037). The ANOVA showed a significant difference between each of the treatments and the abundance of Clostridia (P = 0.0121) yet no significant differences between the treatments was shown with the pairwise comparisons. There were no other significant differences identified in ANOVA or pairwise comparisons for the other identified taxa in the carbonate fraction of the core.

The clay region towards the bottom of the core (215–280 cm), similar to the carbonate region, was dominated by Deltaproteobacteria. The pairwise comparison highlighted a significant difference between treatments As and Af (P =  < 0.0001) and treatments As and B (P =  < 0.0001) but no overall significant difference (P = 0.6336) for the abundance Deltaproteobacteria. There was no significant difference (P = 0.1233) shown for Betaproteobacteria from the ANOVA, however the pairwise comparison indicated a significant difference between treatments As and B (P = 0.0137). The ANOVA revealed a significant difference in the abundance of Actinobacteria (P = 0.0011), Nitrospira (P = 0.0337) and Clostridia (P = 0.0418) between each of the treatments yet no significant differences were observed in the pairwise comparison for these taxa. Moreover, no other significant differences were observed for the other identified taxa in the clay rich sediment.

### Demography of the functionally active sediment microbiome by sedRNA analysis

In addition to identifying the microbes present within the sediment cores under each treatment, sedRNA was purified and sequenced to infer the presence and activity of the microbes at the equivalent sample depths (Table 2). The number of sedRNA sequence reads were orders of magnitude higher for the samples which had been stored for 10 weeks compared to fresh or frozen samples (As and Af). Two blank samples were included for each of the storage conditions to control for any contamination. A high number of sedRNA sequence reads, ascribed to the phylum Firmicutes, was returned from the blanks for condition B samples. The source of this contamination could not be identified, therefore this phylum was therefore removed from all subsequent analysis of this storage condition. sedRNA sequence analysis was performed in the same manner as sedDNA, however after strict quality control filtering, no sedRNA sequence reads remained for sediment horizons of 50, 80, 110, 140, 180 and 230 cm for the sediment that was analysed from conditions As and Af.

**Fig. 5:**
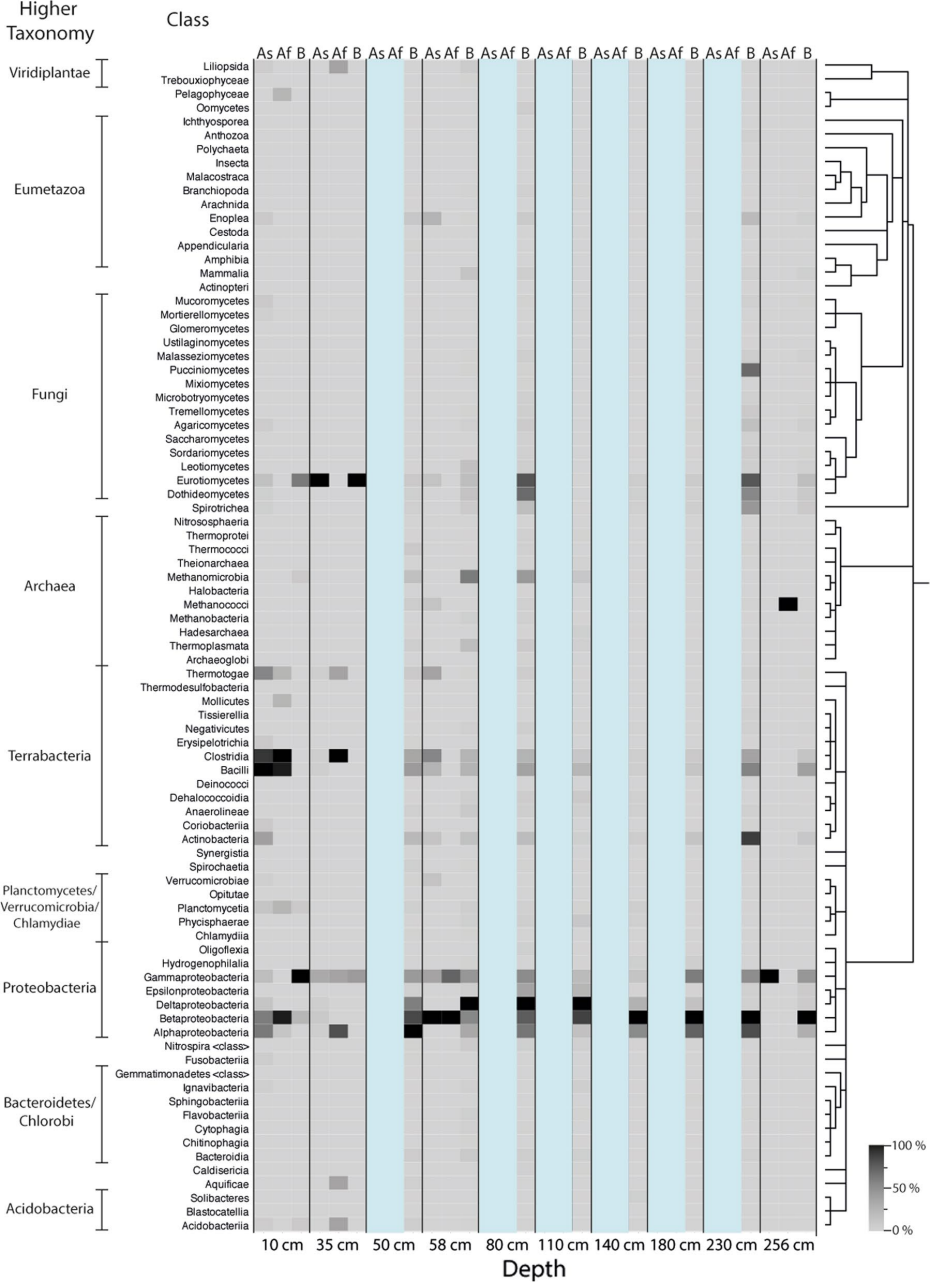
Heatmap representing the taxonomic classifications of sedRNA samples at each condition. Heatmap of the classified taxa at the taxonomic rank of Class displayed using percentage classification. Darker shades denote higher percentages of classified sedRNA sequence reads to that assigned taxa. Condition As—sediment subsampled onsite and sequenced immediately, Af—sediment subsampled onsite, frozen at − 20 °C and sequenced later and B—sediment stored at 4 °C. Taxa are organised by their phylogenetic relationship. Blue bars indicate where no sedRNA sequences were obtained

A heatmap displaying the taxonomic classifications at the taxonomic rank of Class (Fig. 5) shows that *Proteobacteria* is the prominent active Phylum for all sample depths across the storage conditions As, Af and B. However, no individual Class of taxa were dominant across all storage conditions as they were for the sedDNA analysis. There was an abundance of the fungal Class, Eurotiomycetes in conditions As and B at sediment depths of 10 and 35 cm, and this abundance was apparent at depths of 80 and 230 cm for condition B.

Furthermore, the abundance of Methanomicrobia wass exclusively observed in condition B at sediment depths between 10 and 110 cm. At a depth of 35 cm, the condition Af exhibited a high abundance of Clostridia, but condition As did not contain any sedRNA sequence reads assigned to this taxon. Furthermore, sedRNA mapping indicated the Clostridia and Bacilli were active in condition As at 58 cm, but not in condition Af.

At a sediment depth of 10 cm, there was a high abundance of active Alpha- and Betaproteobacteria in condition As, however in condition Af*,* only Betaproteobacteria were observed in high abundance. The majority of Proteobacteria sedRNA in condition B were attributed to Gammaproteobacteria, rather than Alpha- and Betaproteobacteria. Gammaproteobacteria were identified throughout the core in condition B, with Alpha- and Betaproteobacteria observed after a depth of 50 cm. There was a low abundance of Viridiplantae in the upper sediment, above 58 cm from conditions As and Af, likely from plant roots or seed.

Due to an incomplete dataset for the sedRNA samples, it was not possible to perform statistical analysis between each of the treatment conditions.

### Demography of the sediment microbiome at different sediment depths

**Fig. 6:**
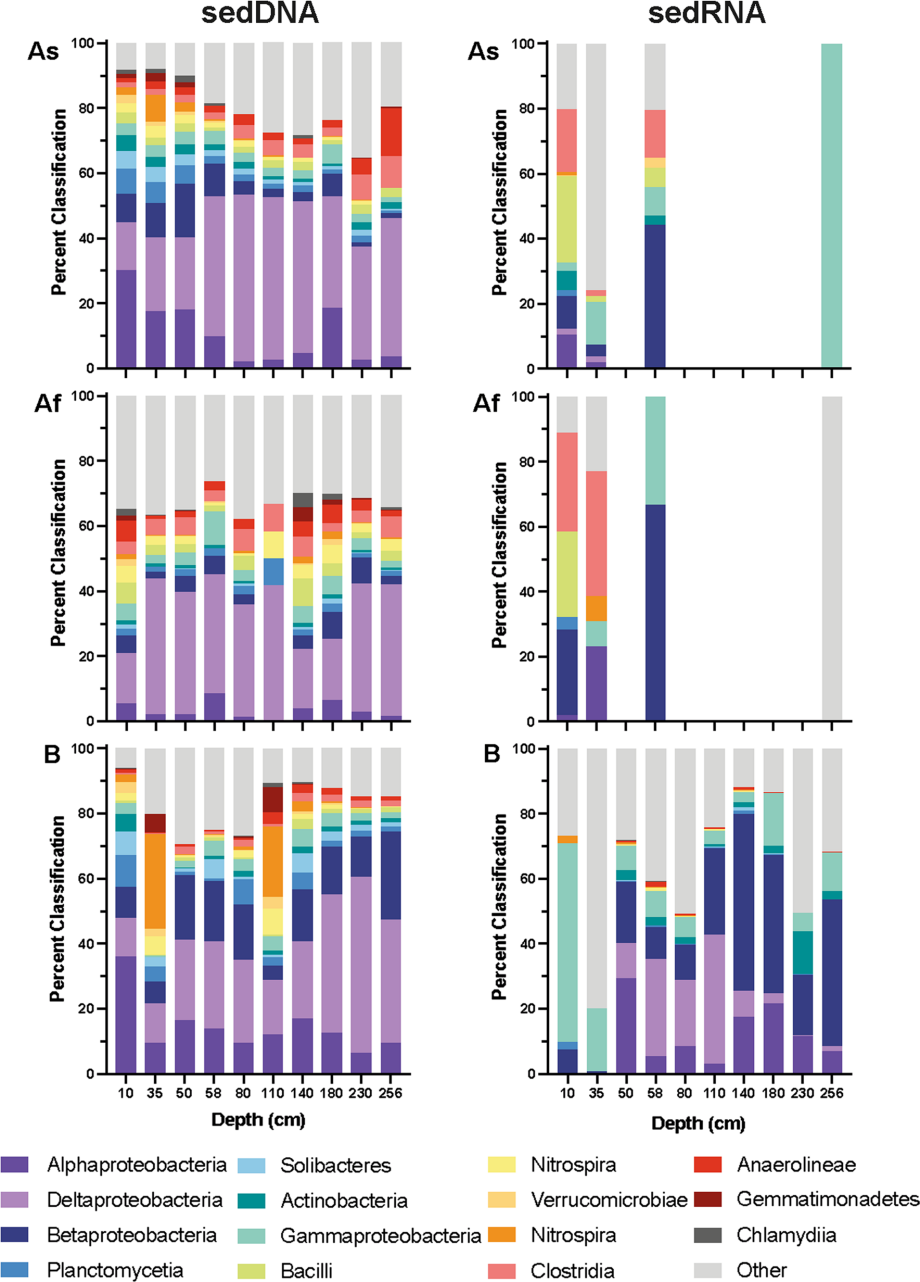
Stacked percentage bar charts comparing the abundant taxa between storage conditions. Stacked bar charts displaying the 15 most abundant taxa classified sedDNA from condition As (Top left). These same taxa are displayed for treatmeant Af (Middle left) and B (Bottom left) for sedDNA analysis and As (Top right)*,* Af (Middle right) and B (Bottom right) for sedRNA analysis. All Firmicutes taxa removed from the RNA treatment B dataset due to contamination of this taxa in the control samples

Similar to the whole-core analysis, comparisons between sample storage conditions were analysed for the 15 most abundant taxa from condition As, these were selected as the baseline against which taxonomic classifications from Core Af and B were compared. To highlight any specific changes in the microbiome, the abundances of these taxa were analysed for each of the individually sampled sediment depths for data from the sedDNA and sedRNA sequencing (Fig. 6).

When analysing the sedDNA dataset, under condition As the Alphaproteobacteria abundance was highest at 30% for sediment from a depth of 10 cm and falls to a low of 2% at 80 cm. Conversely there was a low abundance of Deltaproteobacteria at 10 cm, which rose to over 50% at 80 cm. In shallower sediment, to a depth of 50 cm, there wass approximately 6% of Plactomycetia, which dropped to less than 2% in deeper sediments. Similarly, the abundance of Betaproteobacteria was between 8 and 16% in shallow sediment to a depth of 58 cm. In deeper sediments the abundance of Betaproteobacteria dropped to less than 5%, except at 180 cm where it comprised 7% of the classified taxa. The representation of the other identified abundant taxa, the Solibacteres, Actinobacteria, Gammaproteobacteria, Bacilli, Nitrospira, Verrucomicrobiae, Acidobacteriia, Clostridia, Anaerolineae, Ktedonobacteria and Chlamydiia remained relatively stable throughout the sediment core.

Classified sedDNA comparisons between conditions As, Af and B revealed that the abundance of Alphaproteobacteria identified at a depth of 10 cm in condition Af decreaseed to 5%, compared to 30% in condition As. However, in condition B, where sediment had not been frozen, the abundance of Alphaproteobacteria had increased to 36%, which was comparable to condition As. Through the remaining sediment core, there was a reduction of Alphaproteobacteria at all analysed sediment depths, except 230 cm, for condition Af in comparison to As. Similarly, there was a reduction in the abundance of *Betaproteobacteria* for sediment depths between 10 and 110 cm for condition Af but after 110 cm there was a slight increase in the classified abundance of Betaproteobacteria. However, for condition B, an increase in abundance at all analysed depths, except 35 cm, was observed. Notably at sediment depths of 80, 140, 230 and 256 cm where there was an increase of greater than 10% abundance in Betaproteobacteria for condition B. There was a large variance in the abundance of Deltaproteobacteria at each of the defined sediment depths between storage conditions. At a depth of 35 cm, there was an increase of 19% for condition Af but a reduction of 10% for condition B. At a depth of 50 cm there was an increase of 15% in abundance of samples taken from condition Af, yet only a 2.3% increase in abundance for condition B. At sediment depths between 58 and 140 cm there was reduction in the abundance of Deltaproteobacteria in conditions Af and B, with a 33% reduction observed between treatment As and B at 110 cm. At a depth of 180 cm there was a reduction of 15% for condition Af but an increase in abundance of 8% for condition B. An increase of 4% for condition Af and 19% for condition B was observed at a depth of 230 cm. There is a decrease of 2% for condition Af and 5% for condition B at a depth of 256 cm. There was less than 5% variance in abundance for the taxa Anaerolineae between storage conditions for the sediment core until a depth of 256 cm, where a 13% reduction in abundance was observed for both storage condition Af and B. There was less than 4% variance in abundance for the taxa *Actinobacteria*, Verrucomicrobiae and Chlamydiia throughout the sediment core for each storage condition, and a less than 8% variance in the taxa Gammaproteobacteria, Bacilli, Solibacteres, Plactomycetia, Nitrospira, Clostridia and Ktedonobacteria.

Comparisons between the sedRNA classified in the sediment samples to determine the active microbiome for conditions As, Af and B predominantly revealed an increase in the activity of Alpha-, Beta-, Delta- and Gammaproteobacteria in condition B (Fig. 6 and Additional file 1: Table S2). However, this increase in abundance throughout the sediment core can be attributed to the absence of sedRNA sequence reads for condition As and Af at particular depths. At a sediment depth of 10 cm, there was an 8% reduction in the abundance of Alphaproteobacteria in condition Af and no activity attributed to Alphaproteobacteria for condition B. There was a 16% increase in the abundance of Betaproteobacteria for condition Af at a sediment depth of 10 cm, however there was a 2% reduction observed in condition B. Furthermore, at 10 cm there was a complete reduction of active Actinobacteria from 6% in condition As to 0% for condition Af and B Similarly, at a sediment depth of 58 cm in condition B there was an increase of 22% of active Betaproteobacteria and a reduction of 34%. There was a less than a 1% increase for the taxa Plactomycetia, Solibacteres, Nitrospira, Anaerolineae and Chylamydiia between conditions.

### Principal coordinate analysis of sediment microbiome and depth

Principal Coordinate Analysis (PCoA) of the different storage conditions (Fig. 6) for the sedDNA analysis revealed that there was congruence between conditions As (purple) and Af (blue), especially for samples which were extracted from carbonate sediment, between a depth of 80 and 180 cm. However, there was divergence when comparing these conditions with condition B (green), especially with samples extracted from sediment depths 110 and 180 cm. When comparing samples that were extracted from organic sediment at depths of 10, 35 and 50 cm, there was a greater coherence between samples extracted from conditions Af and B. However, for samples from a depth of 58 cm there was a closer relationship between condition As and B. Samples that were extracted from a depth of 230 cm clustered together for condition As and Af, as well as samples extracted from a depth of 256 cm for condition As and Af, although either cluster is in different positions on the PCoA plot. Samples from the clay sediment at 230 and 256 cm for condition B, clustered closely together but in a different position to condition As and Af.

**Fig. 7:**
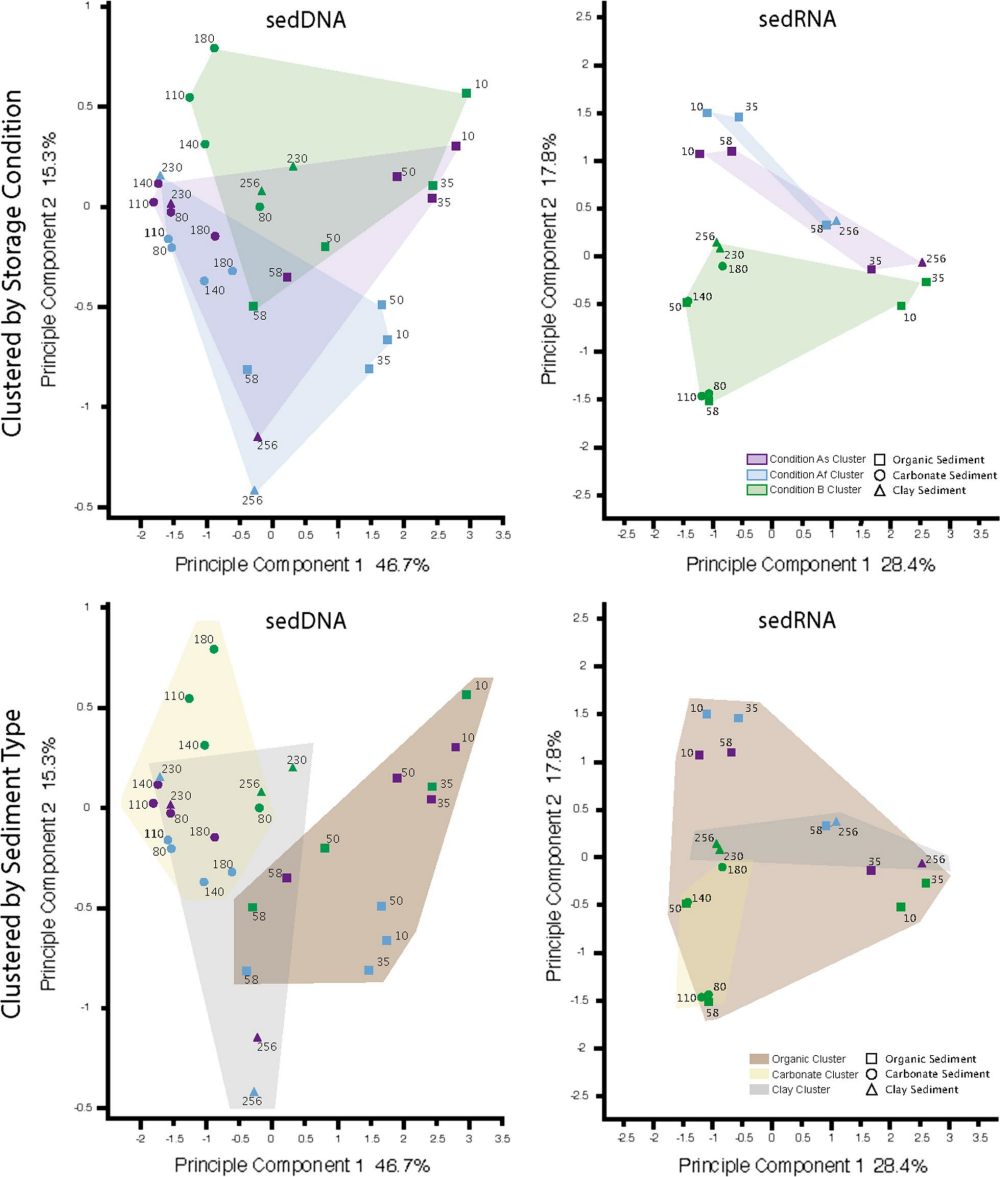
Principal Coordinate Analysis of sedDNA and sedRNA storage conditions. Principal coordinate analysis of the sedDNA and sedRNA analysis performed on condition As, Af and B using MEGAN. Top: Clustered by treatment, purple shading- Condition As, which was subsampled and sequenced on-site. Blue shading—Condition Af, which was subsampled on site and frozen at − 20 °C, sequencing was performed upon return to the laboratory. Green shading—Condition B which was transported back to the laboratory and stored at 4 °C. After 10 weeks, subsampling and sequencing was performed. Bottom: Clustered by sediment type, brown shading encompasses samples from the organic region of the core, beige shading encompasses samples from the carbonate region of the core and grey shading encompasses samples from the clay region of the core. Squares denote sediment that was visually identified as organic, circles denote sediment that was visually identified as carbonate and triangles denote sediment that was visually identified as clay. Sampling depths are shown in cm beside each symbol. Percentage variation displayed for PC1 and PC2

When comparing the PCoA analysis performed on the sedRNA data (Fig. 7) there was minimal overlap in the cluster boundaries between conditions As and Af. With exception of samples extracted for a depth of 10 cm, there was no congruence between samples at the defined sediment depths with these conditions. There was no correlation between condition As or Af and condition B for sedRNA samples. When comparing the sediment composition from which sedRNA samples were extracted, the samples extracted from both carbonate (yellow) and clay (red) sediments clustered within the boundaries of the organic (grey) sediment samples cluster. This was dissimilar to the sedDNA analysis, where despite there being overlaps between the clusters there was distinction between each of the sediment clusters.

## Discussion

Lacustrine sediments are an excellent archive of microbial communities, however in situ subsampling, library preparation and nucleic acid sequencing is not always feasible due to time constraints, the sampling environment or access to the necessary laboratory equipment and sequencing facilities. It is therefore commonplace to transport entire cores or core subsamples back to the laboratory and store them before the relevant analysis can be performed [16]. Guidelines have been proposed how to minimise the effect this may have on the microbiome [10], however, to date the effect of this storage has not been investigated. The Oxford Nanopore MinION sequencing device is suitable for in-situ sequencing and enables accurate and robust analysis of the sediment microbiome [24, 25]. The ability to perform in field sequencing now means that sediment transportation and storage is no longer a compounding factor. To understand the effect of storage on the microbiome we extracted two adjacent sediment cores from an ancient lake site, sub-sampling one core immediately and sequencing the sedDNA and sedRNA on site using the Oxford Nanopore MinION. Additional subsamples were extracted from the same core and stored at − 20 °C. Subsequent sedDNA and sedRNA analysis was performed upon return to the laboratory one week later. The second sediment core was wrapped immediately and analysed on return to the laboratory and after 10 weeks storage at 4 °C.

Here we demonstrate using sedDNA analysis that sediment storage had a significant effect on the abundance on particular taxa throughout the entire core, including Alpha- and Betaproteobacteria, Solibacteres and Bacilli. Furthermore, sediment type (organic, carbonate, clay) also influences how specific taxa are affected when stored. A significant difference was observed between the three conditions for the abundance of Betaproteobacteria detected in the organic and carbonate fractions of the core, but no significant difference was observed in the clay rich regions. Conversely, abundances of Actinobacteria and Nitrospira observed within the clay fraction of the cores were significantly different between the three treatments but not within the predominately organic and carbonate regions. Subsamples that were frozen show the greatest difference in taxonomic classification to samples which were either purified on site or from cores which were stored at 4 °C for 10 weeks. This observation may be ascribed to the disproportionate cryogenic effect on the microbiome when freezing samples, whereby freezing the subsamples increases the lysis efficiency of the DNA purification affecting the observed microbiome [26, 27]. The observed variations corresponding to different sediment types suggests that the chosen storage/treatment method for extracted sediment archives, has important implications for interpreting down-core changes in sedDNA, not just between different paleoenvironmental studies, but within individual archives.

The functionally active microbiome, inferred by sedRNA analysis, displayed divergent clustering when analysed by PCoA, corroborating previous findings on marine sediment that show microbiome functionality changes during storage [15]. The low number of sedRNA sequence reads obtained for the samples which were sequenced on-site, and those immediately frozen and sequenced later is surprising but a potential rationale for these contrasting results is the viable but nonculturable (VBNC) state of sediment bacteria. When in VBNC state, the microbes operate at low metabolic rates and are unculturable until conditions are favourable [28–30]. The process of sediment extraction, shipping and storage maybe sufficient to revive these bacteria from their VBNC state, causing them to have increased metabolic activity which can thus be detected by sedRNA analysis. The vast majority of the sedRNA reads throughout the core from treatment B can be ascribed to Alpha-, Beta-, Delta-, or Gammaproteobacteria. While storage at 4 °C was performed to mimic the originating soil temperature and match conventional laboratory protocols, the exposure to oxygen, or other environmental factors during the coring process may be the cause of an increased metabolic activity from these taxa. Species within the phylum *Proteobacteria* are known psychrophiles which are commonly found in sediments [31–33]. Furthermore, psychrophiles can exhibit a slow growth rate but an increased metabolic activity [34] which may provide justification to why a notable increase in the abundance of Alpha-, Beta-, Delta-, or Gammaproteobacteria is not observed with sedDNA analysis when storing cores at 4 °C.

In a conventional laboratory environment when immediate RNA purification or flash freezing in liquid nitrogen is not possible, RNA stabilising solutions, such as RNAlater (ThermoFisher, UK), are employed to minimise RNA degradation. However, previous studies have demonstrated that RNAlater has a detrimental effect on soil samples, due to their humic acid content [35]. Similar to existing guidelines [10], we recommend nucleic acid purification be performed as soon as possible after core extraction, as storage is shown (Figs. 3, 4) to have a significant effect on the microbiome.

## Conclusions

We conclude that sample storage has a significant effect on the sediment microbiome analysed using sedDNA, especially for Alpha-, Beta- and Deltaproteobacteria species. Furthermore, these significant differences are observed regardless of sediment type. We show that the taxa predominantly affected by storage are Proteobacteria, and recommend on-site purifications are performed to ensure an accurate representation of these taxa in the microbiome. If on-site purifications are not possible, then a test of possible storage conditions should be conducted to identify any negative effects of storage on the observed microbiome. However, if the functionality of the environmental microbiome in the sampled location is required, then on-site purification of the sedRNA is imperative. Whilst RNA stabilisation reagents are available, e.g. RNAlater, these reagents may not be suitable for the sample type and require either contiguous subsampling of the entire core or prior knowledge of the core and horizons of interest which may not be evident at all locations. The Oxford Nanopore MinION sequencing device is suitable for in-situ sequencing of sedDNA and sedRNA enabling accurate and robust analysis of the sediment microbiome, especially when samples cannot be transported internationally. Further investigation into the stability of the microbial structure over months and years, will ascertain if previously acquired and stored sediments can be reliably utilised for sedDNA sequencing.

## Supplementary Information

**Additional file 1.** sedDNA and sedRNA rarefaction curves and abundance changes of selected taxa for each storage condition.

## Data Availability

The datasets generated and analysed during the current study are available in the NCBI SRA repository under BioProject ID PRJNA548524.
