# Supplementary material for: In-situ sequencing reveals the effect of storage on lacustrine sediment microbiome demographics and functionality
Source: Environ Microbiome. 2022 Jan 31;17:5. doi: 10.1186/s40793-022-00400-w (PMC8805238; doi:10.1186/s40793-022-00400-w)
Supplement: Supplementary file 1 — Additional file 1. sedDNA and sedRNA rarefaction curves and abundance changes of selected taxa for each storage condition. [file 40793_2022_400_MOESM1_ESM.docx]

**In-situ sequencing reveals the effect of storage on sediment microbiome demographics and functionality.**

**Supplementary Information**

Richard K. Tennant^1^, Ann L. Power^1^, Norman Sinclair^2^, Sara K. Burton^1^, David A. Parker^1^, Rob Lee^1^, Richard T. Jones^3^ and John Love^1^


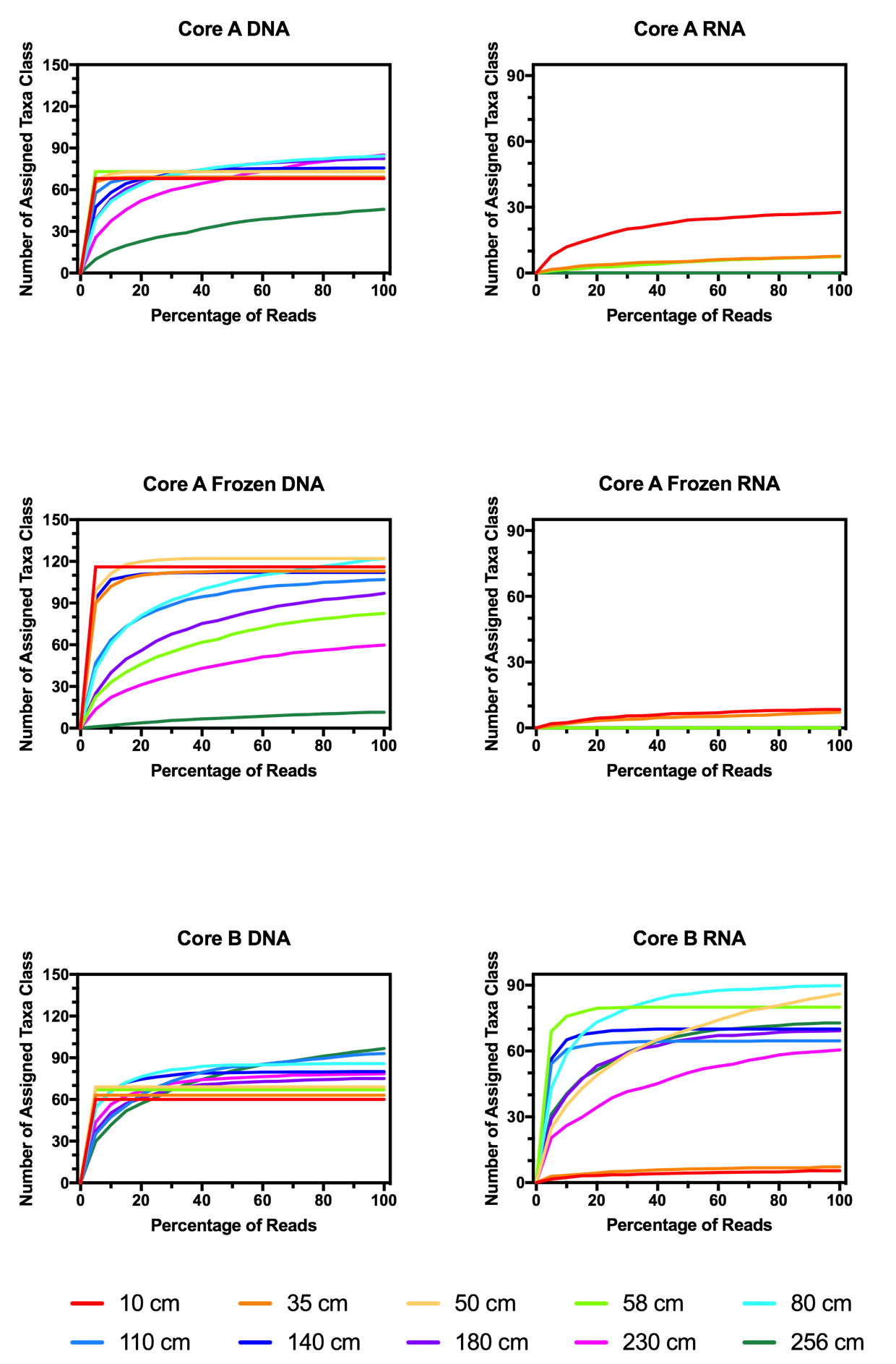


**Supplementary Figure 1 – Rarefaction Analysis of sedDNA and sedRNA sequence data at Class taxonomic level for each of the storage conditions.**


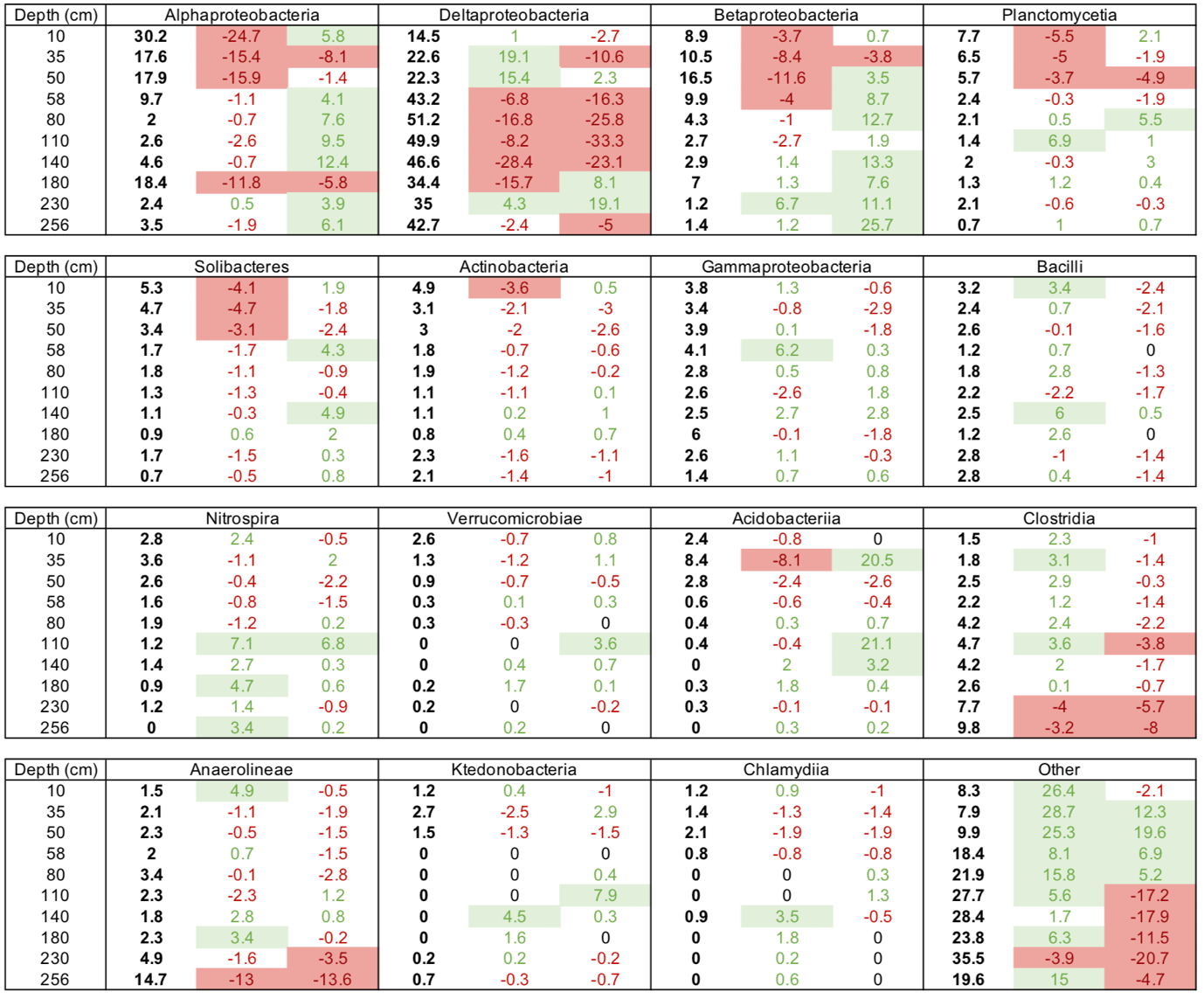


**Supplementary Table 1 - Change in abundance between conditions As, Af and B for sedDNA analysis.**

Displaying percentage classifications for the 15 most abundant taxa identified in condition As.

Column 1. Percentage abundance identified at each sampled sediment depth.

Column 2. Change in percentage abundance for condition Af.

Column 3. Change in percentage abundance for condition B.

Red number/red background indicates percentages that are greater than 4 % lower than condition As. Red number/white background indicates percentages that are between 0 and 4 % lower than condition As. Green number/green background indicates percentages that are greater than a 4 % increase from condition As. Green number/white background indicates percentages that are between 0 and 4 % higher than condition As.


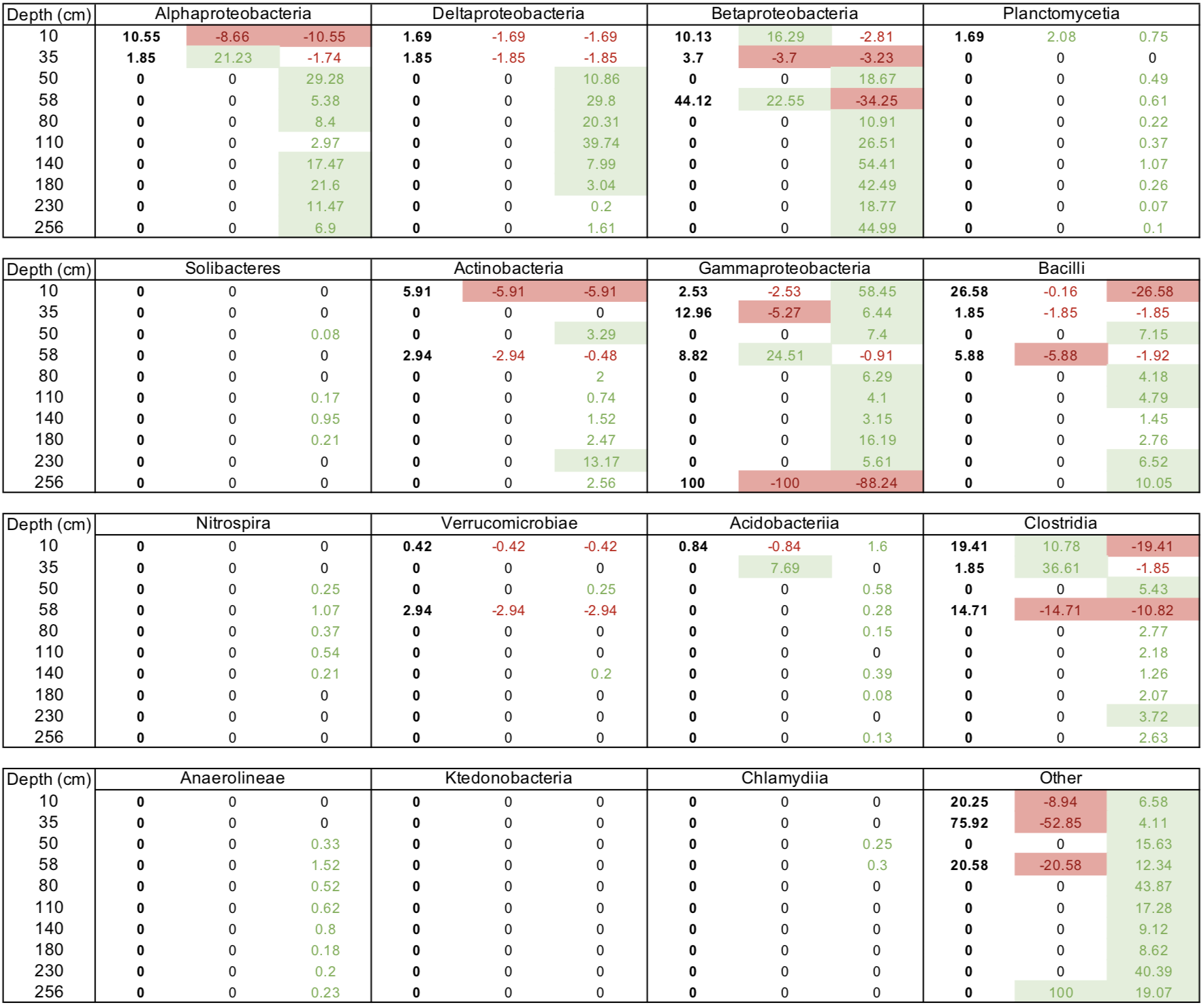


**Supplementary Table 2 - Change in abundance between conditions As, Af and B for sedRNA analysis.**

Displaying percentage classifications for the 15 most abundant taxa identified in condition As.

Column 1. Percentage abundance identified at each sampled sediment depth.

Column 2. Change in percentage abundance for condition Af.

Column 3. Change in percentage abundance for condition B.

Red number/red background indicates percentages that are greater than 4 % lower than condition As. Red number/white background indicates percentages that are between 0 and 4 % lower than condition As. Green number/green background indicates percentages that are greater than a 4 % increase from condition As. Green number/white background indicates percentages that are between 0 and 4 % higher than condition As.
